# Adamantane-Resistant Influenza Infection During the 2004–05 Season

**DOI:** 10.3201/eid1401.070460

**Published:** 2008-01

**Authors:** Mahbubur Rahman, Rick A. Bright, Burney A. Kieke, James G. Donahue, Robert T. Greenlee, Mary Vandermause, Amanda Balish, Angela Foust, Nancy J. Cox, Alexander I. Klimov, David K. Shay, Edward A. Belongia

**Affiliations:** * Marshfield Clinic Research Foundation, Marshfield, Wisconsin, USA; † Centers for Disease Control and Prevention, Atlanta, Georgia, USA; 1 Current affiliation: University of Texas Medical Branch, Galveston, Texas, USA; 2 Current affiliation: Novavax, Malvern, Pennsylvania, USA

**Keywords:** Adamantane resistance, influenza, clinical feature, dispatch

## Abstract

Adamantane-resistant influenza A is an emerging problem, but infections caused by resistant and susceptible viruses have not been compared. We identified adamantane resistance in 47% of 152 influenza A virus (H3N2) isolates collected during 2005. Resistant and susceptible viruses caused similar symptoms and illness duration. The prevalence of resistance was highest in children.

During the past 4 decades, antiviral drug therapy has been a useful strategy for both prophylaxis and treatment of influenza A (*1**,**2*). The most widely used drugs have been 2 adamantane derivatives (amantadine and rimantadine), which are effective for prophylaxis and reduce illness duration if started within 48 hours after symptom onset (*3**,**4*). These drugs block the M2 ion channel protein, preventing viral replication (*5*). From 1997 through 2004, few influenza A (H3N2) isolates were resistant to adamantanes, although resistance among isolates from Asia increased substantially in 2003 (*6*). In the United States, the proportion of influenza A (H3N2) isolates with adamantane resistance was 0.8%–2.2% during this period and 11% in the 2004–05 influenza seasons. However, in the 2005–06 season, >90% of influenza A (H3N2) isolates from patients in 26 states contained a mutation conferring resistance to adamantane drugs (*7*).

Ferret models suggest that virulence is not increased by adamantane resistance in influenza A (H3N2) infections (*8*), although experimental studies with recombinant influenza (H1N1) in mice have suggested that the mortality rate is increased by a double mutation conferring adamantane resistance (*9*). Little is known regarding clinical effects in humans infected with adamantane-resistant influenza viruses. We compared clinical and demographic characteristics of patients infected with either adamantane-susceptible or -resistant strains of influenza A during the 2004–05 season.

## The Study

Study participants were derived from a study of influenza vaccine effectiveness conducted within a 14–Zip code region surrounding Marshfield, Wisconsin, during January–March 2005. Eligible participants included children 6–23 months of age, adults >65 years of age, and persons 24 months–64 years of age with a high-risk medical condition. Research coordinators recruited eligible patients and obtained samples for influenza culture during inpatient and outpatient encounters for acute respiratory illness. Each participant (or parent) completed a short interview form to assess symptoms and onset date, and culture-confirmed patients were contacted again to determine the illness recovery date. The study was approved by the Marshfield Clinic institutional review board, and all participants provided written informed consent.

Influenza virus was isolated by inoculation and incubation in monolayered rhesus monkey kidney cells. Cultures were examined for cytopathic effect, and cultures showing no cytopathic effect were tested twice for hemagglutination titers. Influenza A and B were confirmed by immunofluorescence (Chemicon International, Inc., Temecula, CA, USA). All influenza A isolates were tested for adamantane resistance at the Centers for Disease Control and Prevention (CDC). Procedures for RNA extraction and pyrosequencing have been previously described (*7*). A medical record review was performed to assess any use of antiviral drugs by influenza A patients from 60 days before through 14 days after the enrollment date.

Univariate comparisons were performed, and crude and adjusted prevalence ratios were computed to evaluate the association between adamantane resistance and age, gender, vaccination status, date of clinical encounter, and presence of a high-risk medical condition. Enrollment dates were grouped into 4 consecutive 3-week periods beginning January 3, 2005. The referent period was the second 3-week period when the number of influenza cases peaked. All analyses were conducted with SAS version 9.1 (SAS Institute Inc., Cary, NC, USA).

Influenza virus was isolated in 167 (20%) of 818 cultures from ill cohort members; 153 (92%) were influenza A (H3N2) and 14 (8%) were influenza B (Figure). Influenza A (H1N1) was not isolated from any patients. Adamantane resistance was present in 72 (47%) of 152 influenza A isolates; 1 additional isolate was not characterized. All resistant isolates contained a point mutation resulting in a serine to asparagine change at amino acid 31 (S31N) of the M2 protein. The median age was 43.6 years among patients with resistant isolates and 64.7 years among those with susceptible isolates (p = 0.002). The proportion of patients with adamantane-resistant viruses was significantly higher in 6- to 23-month-old children (70%) compared with adults >65 years of age (39%) (unadjusted prevalence ratio 1.8; 95% confidence interval [CI] 1.2–2.6; p = 0.003).

**Figure:**
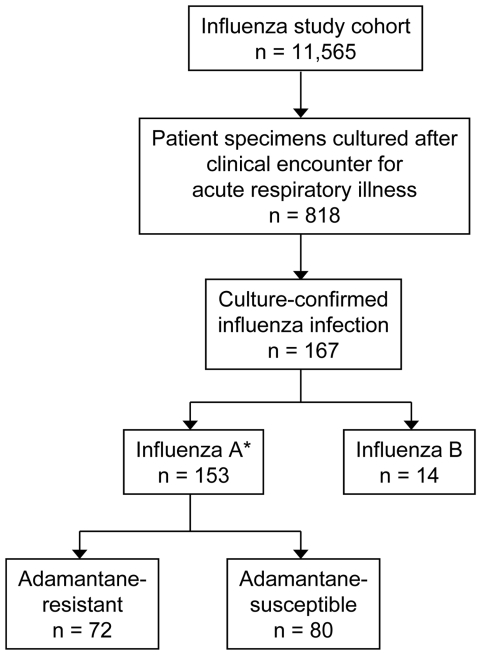
Results of patient recruitment and influenza cultures. *One isolate was not characterized.

Number of symptoms and duration of illness were similar for patients with adamantane-resistant and -susceptible influenza isolates (Table 1). The proportion of patients hospitalized was nearly the same in each group, and the distribution of individual symptoms did not differ significantly. Forty-five patients received antiviral therapy (37 adamantanes, 8 oseltamivir), but only 1 was treated with amantadine before influenza culture was obtained. Clinical features and duration of illness were similar whether or not patients received adamantane therapy. In a multivariable model that included patients of all ages, adamantane resistance was not associated with gender, time of clinical encounter, or influenza vaccination status (Table 2). Children 6–23 months old without a high-risk medical condition were significantly more likely to be infected with adamantane-resistant influenza A than were adults >65 years of age who had a high-risk condition. In a secondary analysis**,** no association was found between adamantane resistance and number of symptoms, duration of illness, or hospitalization.

**Table 1:** Clinical features of patients with adamantane-resistant or -susceptible influenza A, 2004–05 season

Clinical feature	Adamantane-resistant influenza, n = 72	Adamantane-susceptible influenza, n = 80	p value*
Median duration of illness†	12 days	13 days	0.96
No. (%) hospitalized	7 (10)	9 (12)	0.74
No. (%) fully recovered at time of follow-up interview	63 (88)	65 (81)	0.29
Median no. symptoms‡	9	8	0.59
No. (%) receiving antiviral treatment§	15 (21)	31 (39)	0.02
Median time from symptom onset to healthcare encounter	3 days	2 days	0.34
Single symptom, no. (%)‡			
Fever	34 (69)	52 (74)	0.56
Cough	48 (98)	68 (97)	0.78
Headache	41 (84)	53 (76)	0.29
Muscle pain	37 (76)	45 (64)	0.57
Nasal congestion	40 (82)	54 (77)	0.55
Hoarseness	35 (71)	55 (79)	0.37
Fatigue	45 (92)	66 (94)	0.72
Ear pain	16 (33)	24 (34)	0.85
Sore throat	29 (59)	40 (57)	0.82
Difficulty breathing	28 (57)	35 (50)	0.44
Wheezing	25 (51)	42 (60)	0.33
Nausea	16 (33)	27 (39)	0.51
Vomiting	10 (20)	9 (13)	0.27
Diarrhea	12 (24)	15 (21)	0.70
Rash	2 (4)	2 (3)	1.00
Combined symptoms, no. (%)‡			
Fever and cough	33 (67)	52 (74)	0.41
Fever and headache	31 (63)	43 (61)	0.84
Fever, cough, and headache	30 (61)	43 (61)	0.98
Fever and muscle pain	29 (59)	39 (56)	0.71
Fever, headache, and muscle pain	27 (55)	35 (50)	0.58
Fever, headache, muscle pain, and cough	26 (53)	35 (50)	0.74

*χ^2^, Fisher exact, or Wilcoxon rank sum tests performed as appropriate. †Based on 128 influenza A patients who had recovered fully at the time of follow-up interview. ‡Children 6 to 23 months of age were excluded from the numerator and denominator for symptoms. §One patient had received antiviral treatment prior to influenza culture.

**Table 2:** Demographic features of patients with adamantine-resistant or -susceptible influenza A, 2004–05 season*

Variable	No. (%) resistant isolates, n = 72	No. (%) susceptible isolates, n = 80	Unadjusted prevalence ratio (95% CI)	Adjusted prevalence ratio (95% CI)
Age and high-risk status†				
6–23 mo without high risk	20 (74.1)	7 (25.9)	2.0 (1.3–3.0)	2.1 (1.4–3.1)
6–23 mo with high risk	3 (50.0)	3 (50.0)	1.3 (0.6–3.2)	1.4 (0.5–3.5)
2–17 y with high risk	8 (61.5)	5 (38.5)	1.7 (0.9–2.9)	1.4 (0.8–2.4)
18–49 y with high risk	7 (36.8)	12 (63.2)	1.0 (0.5–2.0)	0.9 (0.5–1.9)
50–64 y with high risk	8 (38.1)	13 (61.9)	1.0 (0.5–2.0)	1.0 (0.6–1.9)
>65 y without high risk	7 (46.7)	8 (53.3)	1.3 (0.7–2.4)	1.2 (0.7–2.3)
>65 y with high risk	19 (37.2)	32 (62.8)	Referent group	Referent group
Sex				
Male	37 (50.7)	36 (49.3)	1.1 (0.8–1.6)	1.1 (0.8–1.5)
Female	35 (44.3)	44 (55.7)	Referent group	Referent group
Dates of clinical encounter				
2005 Jan 3–23	10 (47.6)	11 (52.4)	0.9 (0.5–1.4)	0.9 (0.5–1.5)
2005 Jan 24–Feb 13	44 (55.0)	36 (45.0)	Referent period	Referent period
2005 Feb 14–Mar 6	15 (35.7)	27 (64.3)	0.6 (0.4–1.0)	0.6 (0.4–1.0)
2005 Mar 7–25	3 (33.3)	6 (66.7)	0.6 (0.2–1.6)	0.5 (0.2–1.3)
Received 2004–05 influenza vaccine?				
Yes	45 (42.9)	60 (57.1)	0.7 (0.5–1.0)	0.8 (0.6–1.1)
No	27 (57.4)	20 (42.6)	Referent group	Referent group

*CI, confidence interval. †For the multivariate model, a composite variable was created based on age and the presence or absence of a high-risk medical condition as defined by Advisory Committee on Immunization Practices criteria.

## Conclusions

This study provides epidemiologic evidence that the point mutation (S31N) conferring adamantane resistance has not altered influenza A (H3N2) virulence as measured by clinical symptoms and duration of illness. Hospitalization rates were also similar, although the power to detect a difference was low. These results are consistent with prior research in ferrets, which demonstrated that virulence was similar for adamantane-resistant and -susceptible influenza A (H3N2) isolates (*8*). The source population for this study included persons who were eligible for influenza vaccination based on age group or the presence of a high-risk medical condition. Consequently, participants were more likely to experience influenza complications relative to the general population. The absence of any difference in clinical severity in this higher risk population provides some reassurance that adamantane resistance will not affect overall influenza morbidity.

Factors contributing to rapid emergence of adamantane-resistant influenza viruses are not fully known. Resistance has been observed in a variety of outbreak and institutional settings for many years (*10**–**12*) but did not spread into the general population until recently (6,*7*). The proportion of human influenza A (H3N2) isolates with adamantane resistance increased dramatically in People’s Republic of China and Taiwan in 2003 (*6*). However, the degree to which the spread of resistant viruses from Asia contributed to the rapid escalation of adamantane resistance in the United States is uncertain.

The 2004–05 influenza season provided an opportunity to learn more about the epidemiology of adamantane resistance during a period when drug resistance was increasing in Wisconsin but was not yet universal. Children had the highest risk of acquiring an adamantane-resistant influenza infection and are an important reservoir for transmission of influenza to adult household members and the community (*13*). Still, we cannot explain why children were preferentially infected with adamantine-resistant strains in 2004–05. Only 1 patient was treated with amantadine before enrollment. The higher prevalence of resistance in children was therefore not attributable to individual adamantane use. There may have been multiple introductions of adamantane-resistant influenza viruses from other geographic areas during the 2004–05 season. This phenomenon was described in a recent phylogenetic analysis of influenza A (H3N2) isolates from New York state over a 9-year period (*14*). The analysis suggested that viral evolution within epidemic seasons is dominated by random importation of distinct viral strains from other geographic areas.

CDC continues to recommend that adamantane drugs not be used for influenza treatment in the United States. Given the recent emergence of oseltamivir-resistant influenza A and B infections (*15**,**16*), ongoing monitoring of influenza virus susceptibility to adamantanes and neuraminidase inhibitors is essential.

## References

[1] Dolin R, Reichman RC, Madore HP, Maynard R, Linton PN, Webber-Jones J. A controlled trial of amantadine and rimantadine in the prophylaxis of influenza A infection. N Engl J Med. 1982;307:580–4. 7050702 10.1056/NEJM198209023071002

[2] Tominack RL, Hayden FG. Rimantadine hydrochloride and amantadine hydrochloride use in influenza A virus infections. Infect Dis Clin North Am. 1987;1:459–78. 3332798

[3] Younkin SW, Betts RF, Roth FK, Douglas RG Jr. Reduction in fever and symptoms in young adults with influenza A/Brazil/78 H1N1 infection after treatment with aspirin or amantadine. Antimicrob Agents Chemother. 1983;23:577–82. 6859836 10.1128/aac.23.4.577 PMC184704

[4] Reuman PD, Bernstein DI, Keefer MC, Young EC, Sherwood JR, Schiff GM. Efficacy and safety of low dosage amantadine hydrochloride as prophylaxis for influenza A. Antiviral Res. 1989;11:27–40. 10.1016/0166-3542(89)90018-1 2712549

[5] Wang C, Takeuchi K, Pinto LH, Lamb RA. Ion channel activity of influenza A virus M2 protein: characterization of the amantadine block. J Virol. 1993;67:5585–94. 7688826 10.1128/jvi.67.9.5585-5594.1993 PMC237962

[6] Bright RA, Medina MJ, Xu X, Perez-Oronoz G, Wallis TR, Davis XM, Incidence of adamantane resistance among influenza A (H3N2) viruses isolated worldwide from 1994 to 2005: a cause for concern. Lancet. 2005;366:1175–81. 10.1016/S0140-6736(05)67338-2 16198766

[7] Bright RA, Shay DK, Shu B, Cox NJ, Klimov AI. Adamantane resistance among influenza A viruses isolated early during the 2005-2006 influenza season in the United States. JAMA. 2006;295:891–4. 10.1001/jama.295.8.joc60020 16456087

[8] Sweet C, Hayden FG, Jakeman KJ, Grambas S, Hay AJ. Virulence of rimantadine-resistant human influenza A (H3N2) viruses in ferrets. J Infect Dis. 1991;164:969–72. 1940477 10.1093/infdis/164.5.969

[9] Abed Y, Goyette N, Bolvin G. Generation and characterization of recombinant influenza A (H1N1) viruses harboring amantadine resistance mutations. Antimicrob Agents Chemother. 2005;49:556–9. 10.1128/AAC.49.2.556-559.2005 15673732 PMC547263

[10] Hayden FG, Belshe RB, Clover RD, Hay AJ, Oakes MG, Soo W. Emergence and apparent transmission of rimantadine-resistant influenza A virus in families. N Engl J Med. 1989;321:1696–702. 2687687 10.1056/NEJM198912213212502

[11] Degelau J, Somani SK, Cooper SL, Guay DR, Crossley KB. Amantadine-resistant influenza A in a nursing facility. Arch Intern Med. 1992;152:390–2. 10.1001/archinte.152.2.390 1739371

[12] Mast EE, Harmon MW, Gravenstein S, Wu SP, Arden NH, Circo R, Emergence and possible transmission of amantadine-resistant viruses during nursing home outbreaks of influenza A (H3N2). Am J Epidemiol. 1991;134:988–97. 1951297 10.1093/oxfordjournals.aje.a116184

[13] Hurwitz ES, Haber M, Chang A, Shope T, Teo S, Ginsberg M, Effectiveness of influenza vaccination of day care children in reducing influenza-related morbidity among household contacts. JAMA. 2000;284:1677–82. 10.1001/jama.284.13.1677 11015798

[14] Nelson MI, Simonsen L, Viboud C, Miller MA, Taylor J, George KS, Stochastic processes are key determinants of short-term evolution in influenza A virus. PLoS Pathog. 2006;2:e125. 10.1371/journal.ppat.0020125 17140286 PMC1665651

[15] Hatakeyama S, Sugaya N, Ito M, Yamazaki M, Ichikawa M, Kimura K, Emergence of influenza B viruses with reduced sensitivity to neuraminidase inhibitors. JAMA. 2007;297:1435–42. 10.1001/jama.297.13.1435 17405969

[16] Kiso M, Mitamura K, Sakai-Tagawa Y, Shiraishi K, Kawakami C, Kimura K, Resistant influenza A viruses in children treated with oseltamivir: descriptive study. Lancet. 2004;364:759–65. 10.1016/S0140-6736(04)16934-1 15337401

